# Screening Ba_0.9_A_0.1_MnO_3_ and Ba_0.9_A_0.1_Mn_0.7_Cu_0.3_O_3_ (A = Mg, Ca, Sr, Ce, La) Sol-Gel Synthesised Perovskites as GPF Catalysts

**DOI:** 10.3390/ma16216899

**Published:** 2023-10-27

**Authors:** Nawel Ghezali, Álvaro Díaz Verde, María José Illán Gómez

**Affiliations:** MCMA Group, Inorganic Chemistry Department and Institute of Materials of the University of Alicante (IUMA), Faculty of Sciences, University of Alicante, 03690 Alicante, Spain; ghezalinawel34@gmail.com (N.G.); alvaro.diaz@ua.es (Á.D.V.)

**Keywords:** perovskite, cerium, lanthanum, calcium, magnesium, strontium, copper, soot oxidation, GDI engines

## Abstract

Ba_0.9_A_0.1_MnO_3_ (BM-A) and Ba_0.9_A_0.1_Mn_0.7_Cu_0.3_O_3_ (BMC-A) (A = Mg, Ca, Sr, Ce, La) perovskite-type mixed oxides were synthesised, characterised, and used for soot oxidation in simulated Gasoline Direct Injection (GDI) engine exhaust conditions. The samples have been obtained by the sol-gel method in an aqueous medium and deeply characterised. The characterization results indicate that the partial substitution of Ba by A metal in BaMnO_3_ (BM) and BaMn_0.7_Cu_0.3_O_3_ (BMC) perovskites: (i) favours the hexagonal structure of perovskite; (ii) improves the reducibility and the oxygen desorption during Temperature-Programmed Desorption (O_2_-TPD) tests and, consequently, the oxygen mobility; (iii) mantains the amount of oxygen vacancies and of Mn(IV) and Mn(III) oxidation states, being Mn(IV) the main one; and (iv) for Ba_0.9_A_0.1_Mn_0.7_Cu_0.3_O_3_ (BMC-A) series, copper is partially incorporated into the structure. The soot conversion data reveal that Ba_0.9_La_0.1_Mn_0.7_Cu_0.3_O_3_ (BMC-La) is the most active catalyst in an inert (100% He) reaction atmosphere, as it presents the highest amount of copper on the surface, and that Ba_0.9_Ce_0.1_MnO_3_ (BM-Ce) is the best one if a low amount of O_2_ (1% O_2_ in He) is present, as it combines the highest emission of oxygen with the good redox properties of Ce(IV)/Ce(III) and Mn(IV)/Mn(III) pairs.

## 1. Introduction

Nowadays, the accumulation in the atmosphere of pollutants generated by automobile engines is one of the main environmental problems due to their negative effects. During the combustion process, gasoline and diesel engines emit harmful compounds such as carbon monoxide, nitrogen oxides, and particulate matter (PM) that seriously affect the air quality [1]. These primary pollutants react with other atmospheric compounds to form secondary pollutants, such as, among others, ground-level ozone, which stands out for being harmful to plants and causing respiratory issues in humans. GDI engines present high fuel efficiency and low CO_2_ emissions but generate a high amount of PM, especially with a diameter lower than 10 μm, which is the most dangerous one as it can deeply penetrate into the organism (lungs, bloodstream, etc.) [2]. As a result, the European Commission (by the European Green Deal’s Zero Pollution Action Plan [3]) established the 2030 target, which consists of lowering the number of particulate matter with a diameter of 2.5 μm (PM2.5) by at least 44% of 2005 levels. To deal with this problem, gasoline particulate filters (GPFs) are being used in GDI engine vehicles, which must undergo routine regeneration to avoid soot accumulation in the inner channel. On the other hand, three-way catalysts (TWCs) have been employed to remove the gaseous pollutants from gasoline engines since the 1980s. Thus, both TWCs and GPFs are essential after-treatment devices for GDI engines in order to meet the Euro 6 limits [4]. In this context, catalysts for soot removal at low temperatures and at low oxygen (or zero) partial pressures are highly demanded for GPF devices [5]. Until now, noble metal-based catalysts were the most commonly employed formulations for GDI soot removal, especially those composed by Pt. However, noble metals are scarce and expensive [6,7,8], making it mandatory to look for alternative and more economically accessible formulations. Recently, ceria-based mixed oxides have been proposed as promising catalysts due to their oxygen storage properties and the versatility of Ce to modify its oxidation state [9].

In this context, perovskite-type mixed oxides (ABO_3_) are also considered potential catalytic formulations for soot oxidation, as they exhibit intriguing and adjustable physicochemical properties that could be improved by using different strategies, such as, among others, the modification of the composition by the partial substitution of A and/or B cations [10,11,12,13]. In general, transition metals (Fe, Co, Mn, Cr, Cu, V, etc.) are usually located in the B position, while lanthanide and/or alkaline earth metals (Sr, Ca, Ba, etc.) typically occupy the A position [10,14]. Because of the doping with A and/or B cations with different sizes and oxidation states, the redox properties, the generation of oxygen vacancies and the oxygen mobility of perovskites can be greatly improved [10,15]. Additionally, the doping could also modify the electronic structure and, consequently, the semiconductor properties, making these solids good candidates for several catalytic and electrocatalytic applications, such as the CO_2_ reduction reaction (CO_2_RR) [16,17,18], the oxygen reduction reaction (ORR) [19], the oxygen evolution reaction (OER) [20], and some photocatalytic processes [19,21,22]. Thus, in previous studies, the authors determined that the partial substitution of iron by copper in a BaFe_1−x_Cu_x_O_3_ catalyst series (x = 0, 0.1, 0.3, and 0.4) modifies the catalytic performance for soot oxidation under GDI and diesel engine exhaust conditions [23,24]. These perovskites catalyzed the oxidation of soot in both exhaust conditions that are in the presence of NO_2_ (diesel engines) and the 1% O_2_ (“fuel cut” stage of GDI engine exhaust). BaFeO_3_ perovskite was the most active catalyst, whose performance was mainly related to the lattice oxygen mobility, which decreased with copper content.

On the other hand, it is well established that AMnO_3_ perovskites, due to their redox properties related to the electronic configuration of Mn(III) and Mn(IV) [25,26,27], are active catalysts for oxidation reactions. Moreover, Mn(III) presents the Jahn–Teller effect, which is a distortion that provokes some structural defects that generate active sites for oxidation reactions [28,29,30]. Thus, the presence of these two oxidation states, especially an enriched Mn(IV) surface, increases the oxygen mobility and, consequently, the catalytic activity for the oxidation of soot [31]. In fact, in a previous work, the authors used BaMnO_3_ and BaMn_1−x_Cu_x_O_3_ mixed oxides with a perovskite-like structure (obtained by employing various synthesis methods that allow particular chemical and physical properties) as feasible catalysts for GPF systems [31]. The results obtained for soot removal in simulated GDI engine exhaust conditions (i.e., low percentage of oxygen) reveal that, on the one hand, the presence of oxygen vacancies is required to adsorb and activate oxygen, and, on the other hand, a labile Mn(IV)/Mn(III) redox pair is needed to dissociate the adsorbed oxygen. Thus, the coexistence of both properties allows the transport of the activated oxygen towards the soot.

Considering these conclusions, the aim of this work is the synthesis by the sol-gel method of two series of barium manganese perovskite-type mixed oxides in which 10% of barium has been replaced (i.e., Ba_0.9_A_0.1_MnO_3_ and Ba_0.9_A_0.1_Mn_0.7_Cu_0.3_O_3_, where A = Ca, Sr, Mg, Ce, or La). These samples will be tested as catalysts for GPFs to be used for soot removal in simulated GDI engine exhaust conditions.

## 2. Materials and Methods

### 2.1. Synthesis of Catalyst

The sol-gel method adapted to aqueous medium [32,33,34] was used for the synthesis of the two series (Ba_0.9_A_0.1_MnO_3_ and Ba_0.9_A_0.1_Mn_0.7_Cu_0.3_O_3_) of samples. The metal precursors used are the following: barium acetate (Ba(CH_3_COO)_2_, Sigma-Aldrich, St. Louis, MO, USA 99.0% purity), calcium nitrate tetrahydrate (Ca(NO_3_)_2_*4H_2_O, Sigma-Aldrich, 99.0% purity), lanthanum(III) nitrate hydrate (La(NO_3_)_3_*H_2_O, Sigma-Aldrich, 99.0% purity), magnesium nitrate hexahydrate (Mg(NO_3_)_2_*6H_2_O, Sigma-Aldrich, 99.0% purity), cerium(III) nitrate hexahydrate (Ce(NO_3_)_3_*6H_2_O, Sigma-Aldrich, 99.0% purity), strontium nitrate (Sr(NO_3_)_2_, Sigma-Aldrich, 99.0% purity), copper(II) nitrate trihydrate (Cu(NO_3_)_2_*3H_2_O, Panreac, Castellar del Vallès, Spain, 99.0% purity), and manganese(II) nitrate tetrahydrate (Mn(NO_3_)_2_*4H_2_O, Sigma-Aldrich, 99.0% purity). Additionally, citric acid (C_6_H_8_O_7_, Sigma-Aldrich, 99.0% purity) has been employed as a complexing agent (using a citric acid/Ba ratio of 2), and EDTA (Sigma-Aldrich, 98.5% purity) has also been added as a chelating agent (EDTA/Ba = 2) for the synthesis of the BaMnO_3_ reference sample to avoid the precipitation of metal precursors. To obtain the gel, citric acid was dissolved in 40 mL of distilled water at 60 °C, and then the metal precursors, in the same order in which the metals appear in the perovskite formulae, are added. In the case of the BaMnO_3_ reference sample, EDTA is incorporated into the dissolution before the metal precursors, and, finally, citric acid is included. After that, the solution was stirred at 65 °C for 5 h. Throughout the process, the pH was maintained at 8.5 by adding an ammonia solution (Panreac, 30.0 wt%). Then, the gel was dried at 90 °C for 48 h, and the resulting powder was calcined at 850 °C for 6 h.

### 2.2. Characterization

For sample characterization, the following techniques were employed.

The elemental composition was obtained by Inductively Coupled Plasma Optical Emission Spectroscopy (ICP-OES) on a Perkin-Elmer device model Optimal 4300 DV (Waltham, MA, USA). For each experiment, 10 mg of catalyst was dissolved in a mixture of 5 mL of aqua regia and 10 mL of distilled water.

The textural properties were obtained by N_2_ adsorption (at −196 °C) in an Autosorb-6B device (Quantachrome, Anton Paar Austria GmbH, Graz, Austria). Before the adsorption experiments, degassification at 250 °C for 4 h was carried out.

X-ray Diffraction (XRD) was used for determining the crystalline structure, using the XRD patterns recorded (in a Bruker D8-Advance device, Billerica, MA, USA) between 20° and 80° 2θ angles (step rate of 0.4°/min) and using Cu K_α_ (0.15418 nm) radiation.

Surface chemistry composition was obtained by X-ray photoelectron spectroscopy (XPS) in a K-Alpha photoelectron spectrometer device (Thermo-Scientific, Waltham, MA, USA) with an Al K_α_ (1486.7 eV) radiation source. To obtain XPS spectra, the pressure of the analysis chamber was held at 5 × 10^−10^ mbar. The binding energy (BE) and kinetic energy (KE) scales were adjusted by setting the C 1 s transition to 284.6 eV, and the BE and KE values were determined with the peak-fit software of the spectrometer (Thermo Avantage v5.9929).

Temperature-Programmed Reduction with H_2_ (H_2_-TPR) in a Pulse Chemisorb 2705 (from Micromeritics, Norcross, GA, USA) provided by a Thermal Conductivity Detector (TCD) was used to estimate the reducibility of samples. For the tests, 30 mg of sample, heated at 10 °C/min from 25 °C to 1000 °C in a 5% H_2_/Ar atmosphere (40 mL/min), was used. A CuO reference sample was employed for the quantification of H_2_ consumption.

Oxygen Temperature-Programmed Desorption (O_2_-TPD) experiments were performed in a Thermal Gravimetric Mass Spectrometry (TG-MS) device (Q-600-TA and Thermostar from Balzers Instruments (Balzers, Liechtenstein), respectively). Sixteen milligrams of sample (heated at 10 °C/min from room temperature to 950 °C under a 100 mL/min helium atmosphere) was used. Before the experiments, samples were preheated to 150 °C for 1 h for moisture removal. The 18, 28, 32, and 44 *m*/*z* signals were recorded for the quantification of H_2_O, CO, O_2_, and CO_2_ evolved. The amount of oxygen was calculated based on a CuO reference sample.

### 2.3. Activity Tests

The soot oxidation tests (under simulated GDI engine exhaust conditions) were developed on the TG-MS device employed for O_2_-TPD. Thus, 16 mg of a catalyst and soot mixture (soot:catalyst ratio of 1:8, using Printex-U as model soot in loose contact mode) was preheated at 150 °C (1 h) in a 1% O_2_/He mixture (100 mL/min); subsequently, the temperature was increased until 900 °C at 10 °C/min (soot-TPR). Two different reactant mixtures were employed: (i) 1% O_2_/He, which reproduces “fuel cuts” GDI engine exhaust conditions, and (ii) 100% He, which represents regular stoichiometric GDI engine operations [23,24,31].

The soot conversion and the selectivity to CO_2_ percentages were calculated by these equations:

(1) $$\mathrm{Soot}\;\mathrm{conversion}\;\left(\%\right)=\frac{\sum_{0}^{t}CO_{2}+CO}{\sum_{0}^{final}(CO_{2}+CO)}\times 100$$

(2) $${\mathrm{Selectivity}\;\mathrm{to}\;\mathrm{CO}}_{2}\;\left(\%\right)=\frac{{\mathrm{CO}}_{2\;\mathrm{total}}}{{\left({\mathrm{CO}}_{2}+\mathrm{CO}\right)}_{\mathrm{total}}}\cdot 100\;$$

where $\Sigma_{0}^{t}$(CO_2_ + CO) is the amount of CO_2_ and CO generated at time *t*, while $\Sigma_{0}^{final}$(CO_2_ + CO) is the total amount of CO + CO_2_ evolved during the experiment, coming from the oxidation of the total amount of soot.

## 3. Results and Discussion

### 3.1. Characterization

#### 3.1.1. Ba_0.9_A_0.1_MnO_3_ Series

The nomenclature for the mixed oxides is shown in Table 1, along with the specific surface area values (obtained by applying the Brunauer-Emmett-Teller (BET) equation to N_2_ adsorption data), the A metal content (determined by ICP-OES), and some XRD data.

The low surface areas presented in Table 1 suggest, as expected for perovskite-type mixed oxides [10], that all samples present low specific surface areas and extremely small porosity development that, according to K. Akinlolu [35], could be a consequence of the calcination conditions used in the synthesis (850 °C). Note that the calcination temperature used for synthesis was the minimum one needed to obtain the perovskite phase [36] because, if higher temperatures were used, larger crystal sizes would be obtained, thus promoting a decrease in the number of active sites [37]. On the other hand, ICP-OES data confirm that the mixed oxides contain almost all the amount of A metal supplied during the synthesis.

**Figure 1:**
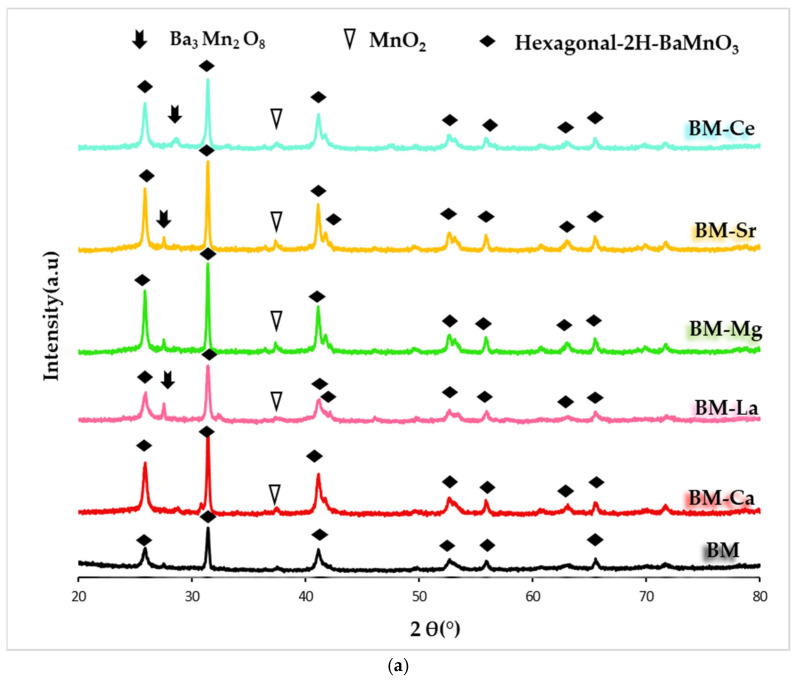

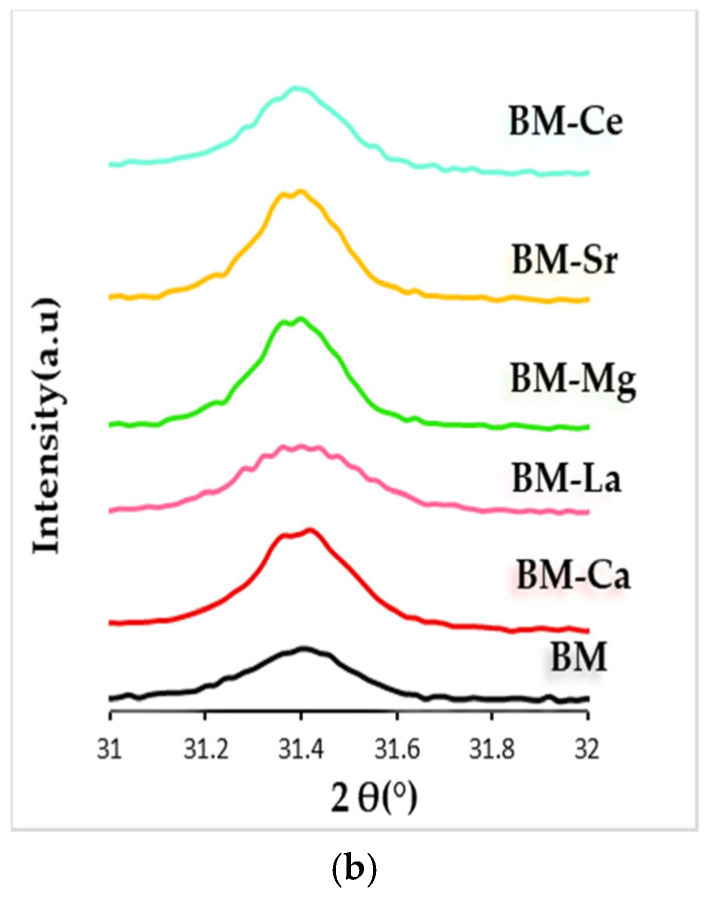
**(a**) XRD profiles of BM-A and BM catalysts, and (**b**) magnification corresponding to the hexagonal 2H-BaMnO_3_ diffraction peak.

Figure 1a shows the XRD patterns of samples, including the raw BaMnO_3_ (BM), obtained also by the sol-gel method. The hexagonal 2H-BaMnO_3_ perovskite structure (PDF number: 026-0168, denoted by the ICDD, the International Centre of Diffraction Data) is the main crystalline phase for all samples [32]. This structure is formed by chains of face-sharing MnO_6_ units rather than by corner-sharing MnO_6_ units [32,36,38]. Note that the partial substitution of the A cation does not cause a significant change to the BaMnO_3_ hexagonal perovskite structure, as the position of the main peak (shown in Figure 1b) and the values of the *a* and *c* cell parameters (included in Table 1) are similar to those shown by BM. However, the intensity of the main XRD peak of the hexagonal perovskite structure is higher for all BM-A samples than for BM perovskite, suggesting that the addition of A metal promotes crystal growth during the calcination step. On the other hand, Ba_3_Mn_2_O_8_ and MnO_2_ (PDF numbers: 073-0997 and 024-0735, respectively) are detected as minority phases. The Williamson-Hall method [39] was used to determine the average crystallite size shown in Table 1, and a decrease is detected after the partial substitution of Ba by A metal, presenting BM-Sr the lowest value.

**Figure 2:**
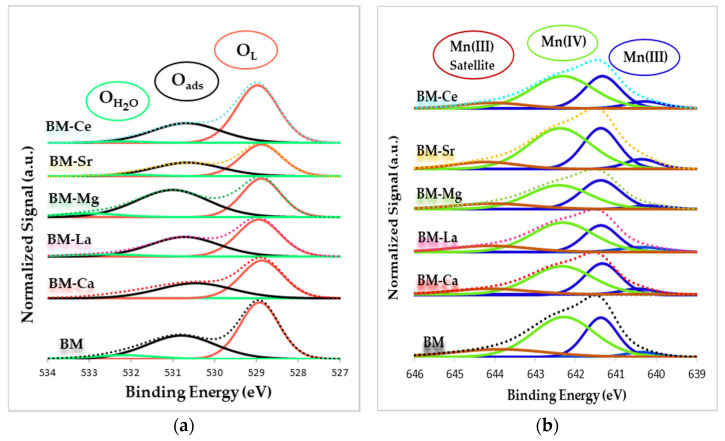
XPS spectra of BM-A and BM catalysts in the (**a**) O 1s and (**b**) Mn 2p_3/2_ core levels regions.

XPS gives information about the species present on the surface and Figure 2 shows the O 1s (a) and Mn 2p_3/2_ (b) XPS spectra for BM and BM-A samples.

In the O 1s XPS spectra, featured in Figure 2a, three different contributions are observed [40,41]: (i) at low binding energies, a sharp peak centered around 529 eV, which is attributed to lattice oxygen (the so-called “O_L_”), (ii) at intermediate binding energies (around 531 eV), a wide peak which corresponds to adsorbed oxygen species (named “O_ads_”), such as surface carbonate (CO_3_^2−^), hydroxyl groups (OH^−^) and peroxide (O_2_^2−^) or superoxide (O_2_^−^) ions, and (iii) a peak at higher binding energies (532 eV) associated to chemisorbed water (labelled as “O_H2O_”). From these XPS spectra, the binding energies for the maximum of the O_L_ and O_ads_ deconvoluted bands have been obtained, which are included in Table 2, and these data indicate that the partial Ba replacement does not significantly affect the binding energy of the deconvoluted peak maxima. The O_L_/(Ba + Mn + A) ratio, calculated using the area under the O_L_ peak and the areas under the signals associated with cations (Table 2), would reveal that surface oxygen vacancies exist if the O_L_/(Ba + Mn + A) XPS ratio is lower than the nominal one (1.5, calculated based on the chemical formulae of the perovskite) [32,40,42]. So, because all the XPS ratios are lower than 1.5, oxygen vacancies exist on the surface of all samples, and the partial replacement of Ba does not substantially change these values. The generation of oxygen surface vacancies [32,36,40], which is required to achieve the positive charge imbalance caused by Mn(III) (see next paragraph), is relevant in the soot oxidation reaction [42] because it allows the creation of the reactive oxygen species.

In the Mn 2p_3/2_ XPS spectra shown in Figure 2b, the following contributions are detected: (i) Mn(III), located at around 641 eV; (ii) Mn(IV), found at approximately 642 eV; and (iii) the Mn(III) satellite peaks at ca 644 eV [32,42,43,44,45]. These spectra reveal that Mn(III) and Mn(IV) coexist on the surface. This fact, previously observed [32,36,45,46] for manganese-based perovskites, indicates that the Mn(II) precursor is being oxidized to Mn(III)/Mn(IV) to achieve electroneutrality on the surface. Note that on the surface of an ideal BaMnO_3_ perovskite (considering the charges of barium (Ba^2+^) and oxygen (O^2−^) ions), Mn(IV) species must be present to preserve the electroneutrality. However, as oxygen vacancies exist on the surface (as already demonstrated by O 1s transition data), Mn(III) should be present to compensate the negative charge deficiency. The Mn(IV)/Mn(III) ratio, calculated with the area of the deconvoluted XPS peaks and included in Table 2, features that Mn(IV) is the predominant oxidation state for all catalysts, as all the Mn(IV)/Mn(III) values are above 1. However, a decrease in the Mn(IV) amount is observed after partial substitution of Ba by A metal, being more significant for BM-Mg. As indicated in the introduction, the presence of both oxidation states is relevant, and it could be expected that, as it was observed in other studies focused on La-Mn and Ba-Mn perovskite-type catalysts [32,38,42,45,46], a surface enriched with Mn(IV) allows improved oxygen mobility, thus enhancing the catalytic activity.

**Figure 3:**
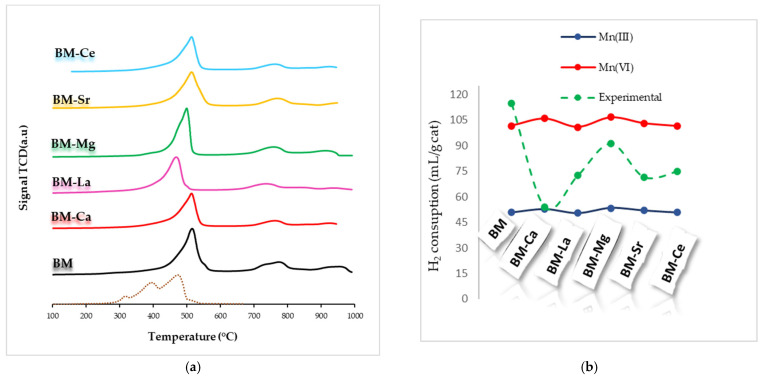
H_2_-TPR profiles of BM-A, BM catalysts, and MnO_2_, used as a reference (**a**) and H_2_ consumption (mL/g of catalyst) (**b**).

Temperature-Programmed Reduction tests using hydrogen as a reducing agent (H_2_-TPR) allow estimating the reducibility and redox properties of samples. The H_2_ consumption profiles for perovskites are determined by the oxidation state and redox properties of the B-site metal [30,45], and the number and sequence of these peaks are strongly dependent on their identity [10,38]. On the other hand, according to the literature, the metal at the A-site typically shows a unique oxidation state and is hardly reduced [10,47]. The H_2_-TPR profile for MnO_2_, used as a reference, presents two overlapping peaks at around 400 and 500 °C that correspond to the reduction of MnO_2_/Mn_2_O_3_ to Mn_3_O_4_ and of Mn_3_O_4_ to MnO, respectively [48]. Usually [30,32,42,45,47], a multiple-step reduction was observed for the manganese-based samples, showing: (i) an intense peak centered between 400–600 °C, corresponding to the Mn(IV) and Mn(III) reduction to Mn(II); (ii) a small peak from 700 °C to 800 °C due to the oxygen species reduction; and (iii) a third peak with a maximum between 900 °C and 1000 °C, corresponding to the Mn(III) to Mn(II) reduction in the bulk [47]. The H_2_-TPR profiles of BM-A samples (Figure 3a) reveal that only for BM-La and BM-Mg, the higher reduction peak appears at lower temperatures than in BM, indicating an improvement in the redox properties. Based on the hydrogen consumption profiles shown in Figure 3a, the experimental hydrogen consumption per gram of sample has been calculated over the temperature range between 450 °C and 600 °C, and these data have been compared in Figure 3b with the theoretical hydrogen consumption calculated assuming a complete reduction of manganese, being as pure Mn(III) (blue line) or pure Mn(IV) (red line). Note that, for BM-A samples, the experimental H_2_ consumptions are placed between both Mn(IV) and Mn(III) nominal values, being closer to Mn(III) for BM-Ca and to Mn(IV) for BM-Mg, as well as for raw BM. Thus, it seems that, as observed on the surface of catalysts (see XPS results), Mn(III)/Mn(IV) oxidation states are present in the bulk of all samples, and the predominant oxidation state is determined by the A metal.

**Figure 4:**
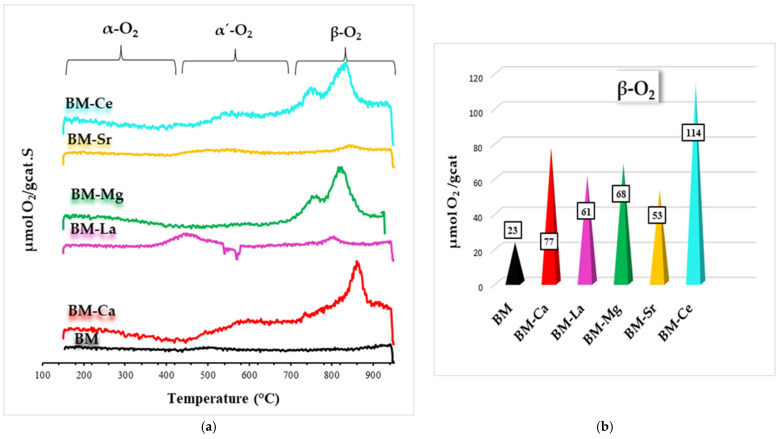
O_2_-TPD profiles (**a**) and β-O_2_ amount (μmol/g cat) (**b**) of BM and BM-A catalysts.

O_2_-TPD tests provide information about the labile oxygen species of the catalysts and about their oxygen mobility [10,36,45], as shown in the profiles displayed in Figure 4a. For perovskites, three oxygen desorption regions are usually detected: (i) the peak located between 150 °C and 350 °C, for the oxygen evolving from the adsorbed on the surface vacancies (called α-O_2_); (ii) the peak appering between 500 °C and 700 °C, corresponding to the oxygen located on surface lattice defects, such as dislocation or grain frontiers (denoted as α′-O_2_); and (iii) the peak over 700 °C, generated by the desorption of lattice oxygen (designed as β-O_2_) due to the reduction of manganese in the perovskite lattice and related to the oxygen mobility through the bulk [10,32,42,45]. Thus, BM-Ca, BM-Ce, and BM-Mg samples evolve oxygen above 700 °C, coming from the perovskite lattice (β-oxygen), which depends on the partial reduction of Mn(IV) to Mn(III) [32,42,46,49], and for BM-Ce, also on the Ce(IV)/Ce(III) redox pair [50]. However, BM-La and BM-Sr do not follow the described trend, as BM-La shows a certain oxygen desorption at a temperature below 700 °C, which corresponds to α’-O_2_, and BM-Sr presents a desorption profile similar to the raw perovskite (BM) without clearly defined peaks. Figure 4b features the amount of β-O_2_ evolved, calculated with the area of the peak between 700 °C and 950 °C (except for BM-La) and CuO as a reference for the quantification. It is observed that, due to the partial replacement of Ba with A metals, the amount of O_2_ released increases, mainly for BM-Ce due to the contribution of the Ce(IV)/Ce(III) redox pair. This finding reveals an increase in the mobility of β-O_2_ due to the presence of A metals, showing BM-Ce the highest value.

#### 3.1.2. Ba_0.9_A_0.1_Mn_0.7_Cu_0.3_O_3_ Series

The nomenclature of catalysts, the BET surface area, the XRD data, and the A and Cu metal contents (obtained by ICP-OES) are reported in Table 3. The ICP-OES data confirm that almost all samples contain the amount of metals (Cu and A) supplied during the synthesis. Additionally, a low surface area is shown for BMC, and the addition of A metal does not significantly affect this parameter. This observation remains consistent with what was previously mentioned for the BM-A series of catalysts.

**Figure 5:**
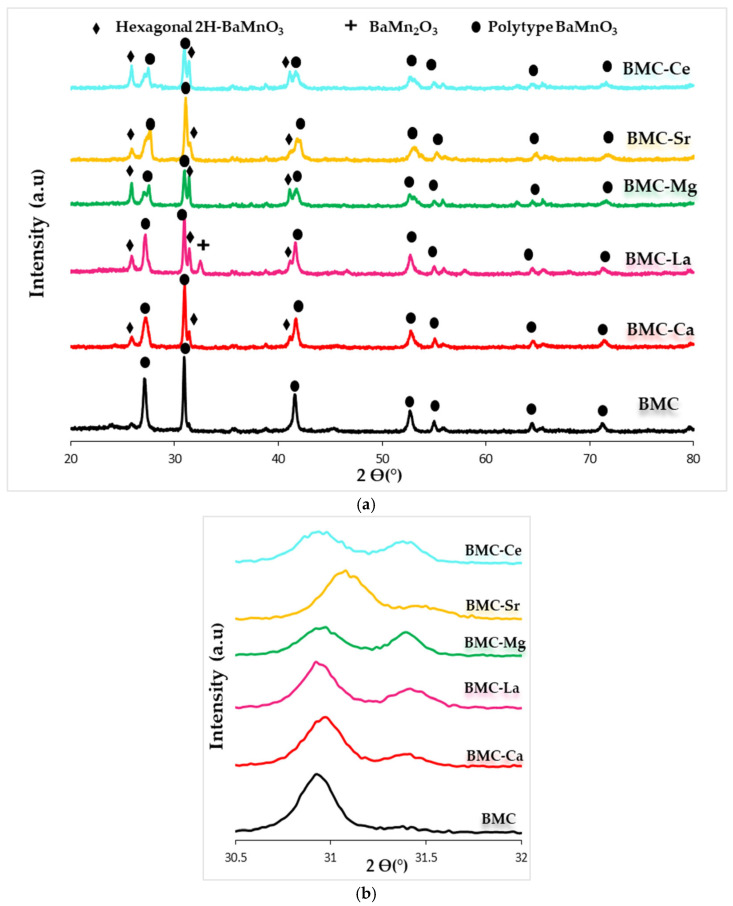
(**a**) XRD profiles of BMC-A and BMC catalysts and (**b**) magnification of the 2Ө diffraction angle region corresponding to main diffraction peak of hexagonal and polytype structure of BaMnO_3_.

Figure 5 features the XRD patterns of all samples. The diffraction peaks for BMC catalyst at 27.0°, 30.9°, 27.5°, 41.5°, 52.9°, 54.8°, 64.3, and 71.0° 2Ө values perfectly match with the BaMnO_3_ polytype perovskite structure [32,36], which is formed because copper partially replace the manganese in the perovskite lattice and causes a different order of the MO_6_ units [51]. However, in BMC-A samples, the polytype structure partially changed back into the hexagonal 2H-BaMnO_3_ structure, thus confirming that A metal has been inserted into the lattice of perovskite. For BMC-La, a peak corresponding to BaMn_2_O_3_ is also detected as a minority phase. The presence of the hexagonal perovskite structure, in addition to the polytype one, could be related to the presence of A metal, which hinders the introduction of copper into the perovskite network. However, as copper species (as CuO) are not clearly detected by XRD, it seems that copper should be inserted into the BM-A perovskites without causing a significant distortion of the hexagonal structure of BaMnO_3_, so this structure seems to be partially preserved for BMC-A. In fact, the coexistence of both hexagonal and polytype structures was previously observed by the authors for under stoichiometric Ba-Cu-Mn perovskites (Ba_0.9_Cu_0.3_Mn_0.7_O_3_ and Ba_0.8_Cu_0.3_Mn_0.7_O_3_) [52]. The transition of the polytype structure to the hexagonal one in BMC-A samples is also evidenced by the decrease in the intensities of the main XRD peak of the former crystalline phase (see values included in Table 3). So, if A metal is present in the catalytic formulation, the crystal growth of the polytype phase seems to be hindered in favour of the formation of the hexagonal structure.

To deeply analyze the partial replacement of Ba by A metal, in Figure 5b, the 2θ diffraction angle between 30.5° and 32.5° (where the main peak of hexagonal and polytype perovskite structures appear, respectively) has been magnified. A slight displacement of the polytype structure main peak to higher diffraction angles with respect to BMC is observed for BMC-Sr catalyst, being less pronounced for BMC-Ca. In this line, Albaladejo-Fuentes and co-workers [53] reported a structural distortion for the Ba_0.9_Sr_0.1_Ti_0.8_Cu_0.2_O_3_ (BTCu-Sr) perovskite due to the partial replacement of Ba(II) by Sr(II). Also, a shift of the main XRD peak was detected by Fu and coworkers [54] due to the presence of the smaller Ca(II) ions in the Ba-site in the Ba_1−x_Ca_x_TiO_3_ catalyst series. Concerning BMC-La, BMC-Ce, and BMC-Mg catalysts, as a change in the position of the polytype BaMnO_3_ structure peak is not clearly detected, it looks as if the A position of the perovskite structure does not include cerium, lanthanum, or magnesium. According to the effect of magnesium on the structure of BaTiO_3_ [53,55], in which Mg(II) was located in the Ti(IV) position, in BMC-Mg, Mg(II) could be replacing Cu and/or Mn instead of Ba. Thus, based on the ionic radius values shown in Table 4, it is suggested that Mg could be incorporated in the B-site (partially replacing Cu and/or Mn) since the ionic radius of Mg(II) is closer to Mn(III) and Cu(II) ionic radii than to Ba(II) radius. However, Ce and La cations should be placed in the A site, since their ionic radii are closer to those of Ba(II), but it seems that this fact happens without a significant distortion of the hexagonal structure. As for the BM-A series, the Williamson-Hall method [39] was applied to determine the average crystallite size of the polytype perovskite phase included in Table 3. The average crystallite size of perovskite decreases for BMC-A samples, achieving the lowest value for BMC-La. On the contrary, the *a* and *c* parameters of BMC-A do not change with respect to the BMC, and only the BMC-Ce catalyst displays a small distortion of the polytype structure.

**Figure 6:**
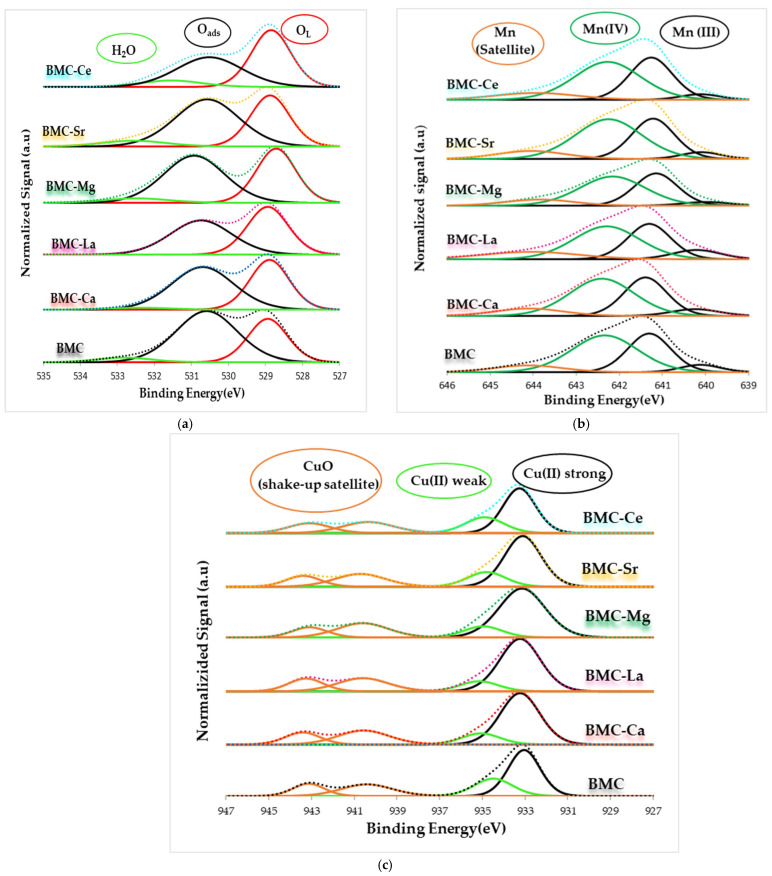
XPS spectra of BMC-A and BMC catalysts in the (**a**) O 1s and (**b**) Mn 2p_3/2_ and (**c**) Cu 2p_3/2_ core level regions.

The XPS spectra for the O 1s, Mn 2p_3/2_, and Cu 2p_3/2_ transitions are shown in Figure 6.

The O 1s spectra of BMC and BMC-A (Figure 6a) feature the three contributions previously described for BM and BM-A, and Table 5 displays some relevant XPS data. Focusing attention on the binding energies corresponding to the maximum of the O_L_ and O_ads_ deconvoluted peaks, BMC-Mg shows the highest chemical shift towards higher binding energies for the O_L_ peak, even though it is very low (0.2 eV). It is important to remember that a shift of the deconvoluted band towards lower binding energies indicates the existence of a richer electronic environment, whereas a displacement towards higher binding energies indicates the opposite. So, the displacement of the O_L_ peak to higher binding energies reveals a poor electronic environment created because of the loss of oxygen from the MnO_6_ octahedra, which, in order to achieve electroneutrality, takes place when Mg(II) occupies the Mn sites. As the O_L_/(Ba + Mn + Cu + A) nominal ratio (1.5) is higher than the XPS one for all perovskites, oxygen surface vacancies exist on all samples. As discussed above for the BM-A series, this fact is caused by the coexistence of surface Mn(III) and Cu(II) [32]. Note that all BMC-A catalysts (except for BMC-Ca) present a slightly higher value of O_L_/(Ba + Mn + Cu + A) ratio than BMC, so it seems that the partial substitution of Ba by A metal in BMC slightly decreases the presence of surface oxygen vacancies.

Figure 6b presents the Mn 2p_3/2_ XPS spectra of BMC and BMC-A samples, where Mn(III) and Mn(IV) signals and Mn(III) satellite contributions were indexed, with Mn(IV) located at 642.4 eV, Mn(III) at 641.2 eV, and the Mn(III) satellite at 644.0 eV [56]. Table 5 provides the binding energy corresponding to the maximum of these decovoluted peaks and, also, the Mn(IV)/Mn(III) ratios. Note that the presence of A metal does not significantly modify the binding energy for Mn(III) and Mn(IV) peaks, being the highest change observed for Mn(III) in BMC-Mg, which, in turn, is low (0.2 eV) and seems to be related to the location of Mg(II) in the Mn site. After doping with A metals, a significant change in the Mn(IV)/Mn(III) ratio is not observed, as the Mn(IV)/Mn(III) ratio decreases from 1.3 to 1.2 for most BMC-A samples, from 1.3 to 1.1 for BMC-La, and is not modified for BMC-Sr. However, as the ratios are higher than 1, Mn(IV) is the main oxidation state on the surface.

The Cu 2p_3/2_ XPS profiles featured in Figure 6c reveal that Cu(II) exists on the surface, as the XPS peaks close to 933.0 eV appear and because the satellite peaks expected for Cu(II) (at 940.0 eV and 943.0 eV) are also present [32]. Additionally, after deconvolution, two contributions are detected at ca. 933.0 eV and 934.5 eV (see Table 5), which can be attributed to Cu(II) with strong (Cu(II)_s_) and weak (Cu(II)_w_) interactions with the perovskites, respectively [23,32,38]. After A doping, an increase in the binding energy corresponding to the maximum of Cu(II)_w_ is detected, which indicates the presence of a poorer electronic environment than in raw BMC caused by the insertion of A metal. In Table 5, the Cu/(Ba + Mn + Cu + A) ratios are lower than the nominal ones (0.15), so it seems that Cu(II) has been inserted into the perovskite structure for all samples. Note that, after the addition of Ce or Ca, the ratio decreases to 0.07, and, for La, it increases to 0.1. Thus, the distribution of copper is only modified with respect to BMC for Ce, Ca, and La metals: Ce and Ca seem to promote the introduction of copper into the structure, and La seems to favour a slightly higher proportion of surface copper [23].

**Figure 7:**
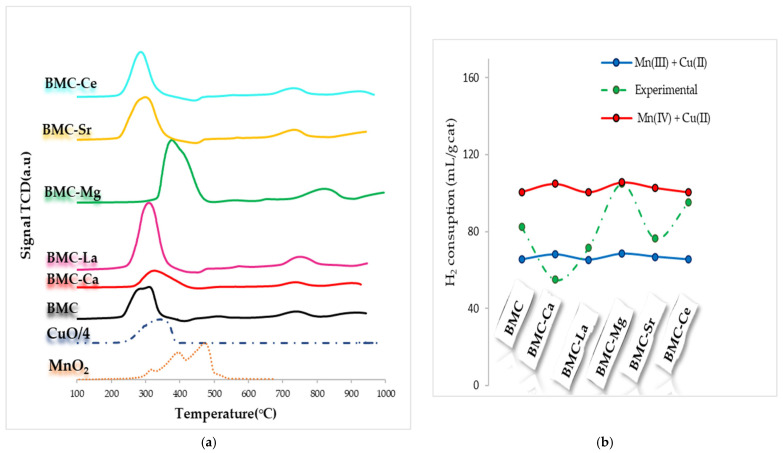
H_2_-TPR profiles of BMC-A and BMC catalysts (**a**) and H_2_ consumption (mL/g of catalyst) (**b**).

H_2_-TPR profiles are shown in Figure 7a, where the corresponding MnO_2_ and CuO profiles (divided by 4 to be comparable with the catalyst profiles) have also been included as references. The H_2_-TPR profile for MnO_2_, as above discussed, features two overlapping reduction peaks at around 400 and 500 °C, corresponding to the MnO_2_/Mn_2_O_3_ reduction of Mn_3_O_4_ and to Mn_3_O_4_ to MnO reduction [45]. CuO displayed at ca. 320 °C a single broad reduction peak due to the reduction Cu(II) to Cu(0). The H_2_-TPR profiles of BMC-A samples suggest that the metal reduction occurs in multiple steps:

- Between approximately 200 and 400 °C, the reduction of Mn(IV) and Mn(III) to Mn(II), and also of Cu(II) to Cu(0), takes place.
- Between 700 °C and 800 °C, a small peak attributed to the desorption/reduction of oxygen species is featured.
- Between 900 °C and 1000 °C, a third peak with very low intensity, corresponding to the bulk Mn(III) reduction, seems to be present.

A decrease in the temperature for the reduction of Mn(IV)/Mn(III) to Mn(II) and of Cu(II) to Cu(0) in the H_2_-TPR profiles of BMC-A (Figure 7a) is exclusively detected after Ce doping. In fact, after Mg doping, an increase in temperature is detected, which could be related to the different location of Mg in this sample. The experimental hydrogen consumption per gram of catalyst, determined between 200 °C and 450 °C using the hydrogen consumption profiles shown in Figure 7a, has been compared with the theoretical hydrogen consumption determined considering the total reduction of manganese and copper (as Mn(III) + Cu(II) in the blue line or Mn(IV) + Cu(II) in the red line) in Figure 7b. For BMC-A samples, the experimental H_2_ consumptions are between the nominal ones for Mn(IV) + Cu(II) and Mn(III) + Cu(II), corresponding to BMC-Mg and BMC-Ce being closer to Mn(IV) + Cu(II), while for BMC, BMC-Ca, BMC-La, and BMC-Sr are closer to Mn(III) + Cu(II). Thus, it appears that both Mn(III) and Mn(IV) exist in the bulk of perovskites, as detected by XPS on the surface.

**Figure 8:**
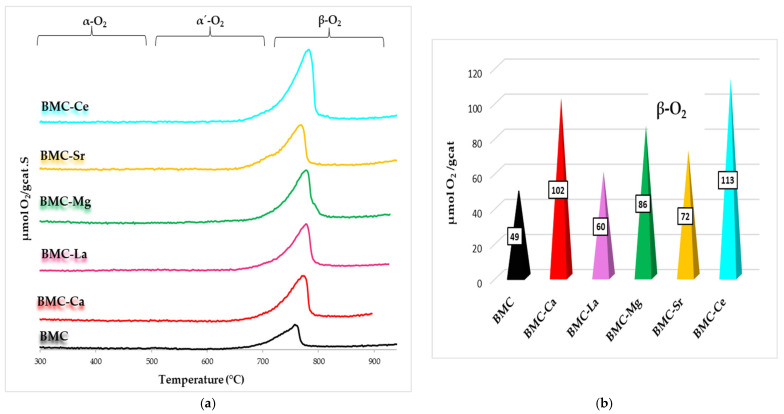
O_2_-TPD profiles of BMC and BMC-A catalysts (**a**) and β-O_2_ amount emitted during O_2_-TPD experiments (**b**).

O_2_-TPD tests were also performed, and the obtained profiles are illustrated in Figure 8. It is observed that, as previously described for the BM-A series, the catalysts exclusively evolve β-O_2_, which is linked to the reduction of Mn(IV) to Mn(III) and of Cu(II) to Cu(I) [57,58,59,60,61] and, for BMC-Ce, also to the reduction of Ce(IV) to Ce(III) [50]. After A metal is included in the formulation of samples, a shift towards higher temperatures is detected for the temperature of the maximum oxygen emission. This fact is directly related to the A-O bond energy, which is expected to be higher than the Ba-O bond energy, as Ba(II) presents a larger ionic radius than the A metal (see Table 4). As shown in Figure 8b, the partial substitution of Ba causes an increase in the amount of β-O_2_ released, which is evidence of the improved mobility of the bulk oxygen, according to the following trend: BMC-Ce > BMC-Ca > BMC-Mg > BMC-Sr > BMC-La > BMC. This finding seems to be related, in general terms, to the reducibility of the samples, as BMC-Ce is the most reducible catalyst and has also evolved the highest amount of oxygen.

In summary, by comparing the characterization results for the two series of A-doped catalysts (BM-A and BMC-A), it seems that:

(i) The hexagonal structure is favoured in the presence of A dopant, as it is the main phase detected for BM-A, and the polytype structure detected in the BMC sample (formed by distortion of the hexagonal perovskite due to copper insertion into the lattice) is not favoured in BMC-A perovskites, presenting a mixture of the two structures. Also, Mn(IV) and Mn(III) coexist on the surfaces of all samples.
(ii) Oxygen vacancies are present on the surfaces of all perovskites.
(iii) The partial substitution of Ba in BM and BMC enhances the reducibility and the lattice oxygen mobility, making Ce the most efficient A metal due to the contribution of the Ce(IV)/Ce(III) redox pair.

### 3.2. Catalytic Activity

#### 3.2.1. BM-A Series

**Figure 9:**
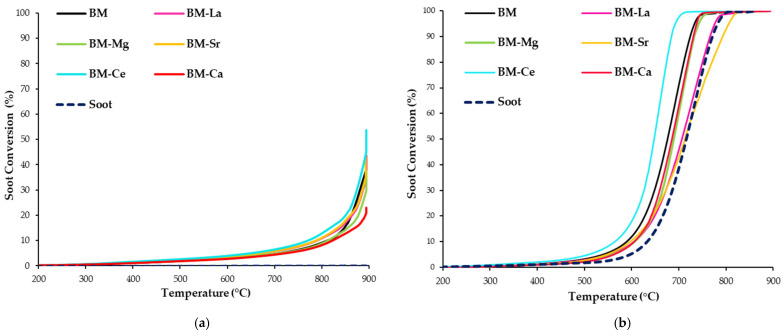
Soot-TPR conversion profiles as a function of temperature of BM and BM-A catalysts in 100% He (**a**) and in 1% O_2_/He (**b**).

To assess the role of perovskites as GPF catalysts to be used for soot removal in simulated GDI engine exhaust conditions, soot-TPR tests in the two gaseous mixtures previously described (0% and 1% O_2_ in He) were carried out [23,24,31], being the soot conversion profiles featured in Figure 9. Table 6 contains the T_10%_ and T_50%_ values, that are the temperatures for the 10% and 50% of soot conversion, respectively, and the selectivity to CO_2_ during the reaction.

Note that almost all samples catalyze the soot oxidation reaction, as most of the profiles are shifted to lower temperatures with respect to the uncatalyzed reaction (soot in Figure 9) in the two atmospheres tested. The soot conversion in the 100% He begins to be significant at above 700 °C (Figure 9a), while in the 1% O_2_, the soot conversion starts being relevant at around 450 °C (Figure 9b). So, as previously concluded [23,31], soot oxidation is improved in the presence of oxygen in the gaseous mixture used for the reaction. Thus, in the He atmosphere, soot oxidation does not occur in the absence of a catalyst, as there is no oxygen available. However, in the presence of perovskites, the reaction takes place with the oxygen coming from the bulk of mixed oxides (β-O_2_), whose emission was promoted by the Mn(IV) to Mn(III) reduction [31], as well as by the Ce(IV) to Ce(III) reduction in the case of the BM-Ce catalyst [50]. In the 1% O_2_ atmosphere, BM-Ca, BM-Ce, and BM-Mg show a higher soot conversion than BM, giving BM-Ce the best catalytic performance.

Thus, the data in Table 6 reveal that the partial replacement of Ba by A metal in BM perovskite improves the catalytic performance for soot removal in simulated GDI engine conditions, as T_10%_ and T_50%_ values decrease with respect to BM for most A metals. BM-Ce is the best catalyst in the two conditions tested, as it presents the lowest T_10%_ and T_50%_ values. This sample presents the highest oxygen mobility due to the contribution of Ce(IV)/Ce(III) along with the Mn(IV)/Mn(III), which enhances the redox reaction and the oxygen emission, which is directly involved in the soot oxidation. Additionally, oxygen vacancies exist on the surface (as O_L_/(Ba + Mn + Cu) XPS ratios are lower than 1.5) that work as active sites where the oxygen from the gas phase is activated and participates in the soot oxidation reaction. So, the more efficient activation of oxygen on the active sites and the higher release of oxygen from the catalyst make BM-Ce the most active sample of the BM-A series.

Finally, regarding CO_2_ selectivity, all BM-A catalysts improve this parameter with respect to BM, so these catalysts seem to be also promising for CO oxidation reactions.

#### 3.2.2. BMC-A Series

**Figure 10:**
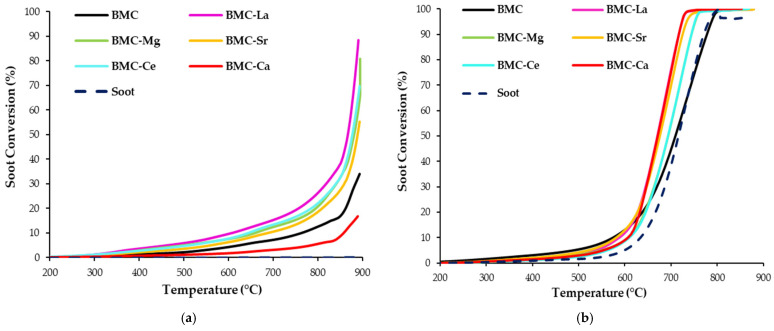
Soot conversion profiles as a function of temperature of BMC and BMC-A catalysts in: (**a**) 100% He and (**b**) 1% O_2_/He.

In order to determine the impact of A metal doping (A = La, Mg, Sr, Ce, Ca) on the catalytic performance of BMC samples for soot removal, as for the BM-A series, soot-TPR tests have been developed using conditions similar to those of GDI engine exhaust. The soot conversion profiles are displayed in Figure 10a,b, and Table 7 summarizes the temperatures needed to achieve 10% and 50% soot conversion, respectively, as well as the selectivity to CO_2_.

First, focusing on the results obtained using 100% He (Figure 10a), all perovskites are active for soot oxidation since the uncatalyzed reaction (denoted as soot in Figure 10) does not take place as there is no oxygen available for the reaction. Note that, after doping BMC with A metal, all the soot conversion profiles are shifted to lower temperatures with respect to BMC. However, the catalytic activity is notably lower than in the 1% O_2_ atmosphere, as the oxygen involved in soot oxidation only comes from the bulk of samples. The BMC-La sample exhibits the lowest T_10%_, probably due to the highest amount of copper on the surface (see Table 5), which is active for soot oxidation [33]. According to the literature, this surface copper species are present as BaO_x_–CuO_x_ phases, forming Cu–O–Ba units in the interface between CuO and the perovskite [24]. As the Cu–O bond energy is lower than that of the Mn–O bond, the release of oxygen from Cu–O–Ba units is easier, thus allowing a high oxygen release rate that improves the catalytic performance for soot oxidation [62]. Aneggi et al. [5], using ceria-zirconia Cu-based catalysts, also detected an improvement in the catalytic activity for soot removal in the absence of oxygen due to the well-known high Oxygen Storage Capacity (OSC) of these mixed oxides. In the 1% O_2_/He reactant mixture, the addition of A metal also enhances the catalytic activity for soot oxidation, and T_50%_ values for BMC-A samples are lower than those corresponding to the raw BMC. In these conditions, BMC-Ca and BMC-La are the most active formulations, as these catalysts increase the amount of oxygen evolved (according to O_2_-TPD results) and present more oxygen vacancies (that allow the activation of oxygen from the gas phase) and surface copper species with a poorer electronic environment than raw BMC (see XPS results); this gives BMC-La the highest proportion of surface copper. In this sense, W.Y. Hernández et al. [63] also found that the use of La_0.6_Sr_0.4_FeO_3_ and La_0.6_Sr_0.4_MnO_3_ allows a notable decrease in the light-off temperatures in the 1% O_2_/He atmosphere. On the other hand, according to other papers [24,46], the CO_2_ selectivity values are higher when oxygen is present in the reaction atmosphere, and the generation of CO_2_ is highly favoured by A doping, being around 90% for all samples. Thus, as well as the BM-A series, these samples are promising catalysts for the CO oxidation reaction.

Finally, by comparing the T_10%_ and T_50%_ values of the best catalysts of the two series analyzed (BM-Ce and BMC-La, selected because they featured the highest selectivity to CO_2_), it could be concluded that:

(i) In 100% He, BMC-La is the most active catalyst, as T_10%_ is lower than the observed for BMC-Ce (611 °C and 646 °C, respectively), mainly because more copper (as BaO-CuO species) is present on the surface of BMC-La than on BMC-Ce (Cu/(Ba + Mn + Cu + A) ratios are 0.10 and 0.07, respectively).
(ii) In 1% O_2_ in He, the best catalyst is BM-Ce, as it presents a lower T_50%_ value than BMC-La (641 °C and 671 °C, respectively) and a similar CO_2_ selectivity (around 90% in both catalysts). BM-Ce is the sample with the highest oxygen mobility and the best redox properties due to the participation of the Ce(IV)/Ce(III) redox pair as well as a high amount of oxygen vacancies on the surface.

So, according to the previous discussion, the role of copper seems to be relevant if the oxygen involved in the soot oxidation comes from the perovskite (i.e., in 100% He), and BMC-La is the most active catalyst as it presents the highest fraction of copper on the surface. However, if soot is oxidized using the oxygen present in the reaction atmosphere (i.e., in 1% O_2_ in He), the presence of copper in the perovskite composition seems not to be significant, as the most active catalyst is BM-Ce. Note that the BM-Ce sample presents a higher fraction of surface Ce(IV) than BMC-Ce (see data in Appendix A), so it has better redox performance.

## 4. Conclusions

In this paper, Ba_0.9_A_0.1_MnO_3_ (BM-A, A = Mg, Ca, Sr, Ce, La) and Ba_0.9_A_0.1_Mn_0.7_Cu_0.3_O_3_ (BMC-A, A = Mg, Ca, Sr, Ce, La) perovskite-type mixed oxides were prepared, characterized, and used for soot removal by oxidation in simulated GDI engine exhaust conditions. Considering the results discussed above, the following conclusions can be drawn:

- The hexagonal structure is favoured in the presence of A metal, as it is the main phase detected for BM-A, and the polytype structure found in the BMC sample (formed by distortion of the hexagonal perovskite due to copper insertion into the lattice) is not favoured in BMC-A perovskites that present a mixture of the two structures.
- On the surface of all perovskites, coexisting Mn(IV) and Mn(III) and oxygen vacancies are present.
- The partial substitution of Ba in BM and BMC enhances the reducibility and the lattice oxygen mobility, and Ce is the most efficient due to the contribution of the Ce(IV)/Ce(III) redox pair.
- Almost all samples are active as catalysts for soot removal by oxidation, as most of the conversion profiles are shifted to lower temperatures in the presence of perovskites in the two atmospheres tested (0% and 1% O_2_ in He).
- The soot conversion is notably lower in the absence of O_2_ than in the 1% O_2_ atmosphere, as the oxygen available for soot oxidation exclusively comes from the bulk of samples. In these conditions, BMC-La is the most active catalyst due to its highest proportion of copper on the surface (as Ba-O-Cu species).
- In 1% O_2_ in He, BM-Ce is the best catalyst as it presents a high amount of oxygen surface vacancies, the highest oxygen mobility, and the best redox properties due to the participation of the Ce(IV)/Ce(III) pair along with the Mn(IV)/Mn(III) pair that promote the O_2_ emission from perovskite, which is directly involved in the soot oxidation.
- The role of copper seems to be relevant only if the oxygen used for the soot oxidation exclusively comes from the perovskite (i.e., in 100% He), as BMC-La, which presents the highest fraction of surface copper, is the most active catalyst. On the contrary, if soot is oxidized using the oxygen present in the reaction atmosphere (i.e., in 1% O_2_ in He), the presence of copper in the perovskite composition is not significant, as the most active catalyst is BM-Ce because it presents a higher fraction of surface Ce(IV) than BMC-Ce and, consequently, a better redox performance.

## Figures and Tables

**Table 1:** Nomenclature, XRD data, metal content and BET surface area of BM-A catalysts.

Nomenclature	Molecular Formula	Specific Surface Area (m^2^/g)	A (wt%)	Intensity (a.u) ^a^	Average Crystal Size (nm)	Cell Parameters (Å) ^b^	
						*a*	*c*
**BM**	BaMnO_3_	3	-	1154	46	5.7	4.9
**BM-Ca**	Ba_0.9_Ca_0.1_MnO_3_	11	1.6	1913	25	5.7	4.8
**BM-La**	Ba_0.9_La_0.1_MnO_3_	7	1.1	1562	28	5.7	4.8
**BM-Mg**	Ba_0.9_Mg_0.1_MnO_3_	7	4.2	2382	28	5.7	4.8
**BM-Sr**	Ba_0.9_Sr_0.1_MnO_3_	5	4.8	2382	18	5.6	4.9
**BM-Ce**	Ba_0.9_Ce_0.1_MnO_3_	10	1.3	1913	22	5.5	5.0

^a^ Corresponding to the main XRD hexagonal perovskite peak, ^b^ Calculated using the main XRD hexagonal perovskite peak.

**Table 2:** XPS data of BM and BM-A catalysts.

Catalyst	B.E Max Mn(III) (eV)	B.E Max Mn(VI) (eV)	B.E Max O_L_ (eV)	B.E Max O_ads_ (eV)	$\frac{O_{L}}{\left(Ba\;+\;Mn\;+\;A\right)}$ (Nominal = 1.5)	$\frac{Mn\left(IV\right)}{Mn\left(III\right)}$
**BM**	641.4	642.3	528.9	530.8	1.0	1.7
**BM-Ca**	641.3	642.3	528.9	530.5	1.0	1.6
**BM-La**	641.4	642.3	529.0	530.7	1.0	1.6
**BM-Mg**	641.4	642.3	528.9	531.0	1.0	1.1
**BM-Sr**	641.4	642.4	528.9	530.7	1.1	1.4
**BM-Ce**	641.4	642.4	529.0	530.7	1.0	1.4

**Table 3:** Nomenclature, XRD data, A and Cu metal contents, and BET surface area of BMC-A catalysts.

Nomenclature	Molecular Formula	A (wt%)	Cu (wt%)	BET (m^2^/g)	2 Ө (°) ^a^	Intensity (a.u) ^a^	Average Crystal (nm)	Cell Parameters (Å) ^b^	
								*a*	*c*
**BMC**	BaMn_0.7_Cu_0.3_O_3_	-	8.0	3	30.9	2448	30.7	5.8	4.3
**BMC-Ca**	Ba_0.9_Ca_0.1_Mn_0.7_Cu_0.3_O_3_	2.0	9.8	7	31.0	1154	29.3	5.7	4.3
**BMC-La**	Ba_0.9_La_0.1_Mn_0.7_Cu_0.3_O_3_	5.4	9.8	7	30.9	2064	18.6	5.8	4.2
**BMC-Mg**	Ba_0.9_Mg_0.1_Mn_0.7_Cu_0.3_O_3_	1.0	9.6	3	30.9	1246	25.9	5.8	4.3
**BMC-Sr**	Ba_0.9_Sr_0.1_Mn_0.7_ Cu_0.3_O_3_	3.9	9.1	9	31.1	1913	25.0	5.8	4.3
**BMC-Ce**	Ba_0.9_Ce_0.1_Mn_0.7_ Cu_0.3_O_3_	2.1	9.2	6	30.9	1441	22.4	5.6	4.3

^a^ Corresponding to the main peak of BaMnO_3_ polytype structure, ^b^ Calculated using the main diffraction peak of BaMnO_3_ polytype structure.

**Table 4:** Ionic radii of cation metals using the Goldshmidt correction [16,31,35].

Metals	Ba(II)	Ca(II)	Mg(II)	La(III)	Ce(IV)	Ce(III)	Sr(II)	Cu(II)	Mn(IV)	Mn(III)
Ionic radii (pm)	146.4	115.5	65.0	107.3	90.6	105.2	129.9	73.0	53.0	65.0

**Table 5:** XPS data of BMC-A and BMC catalysts.

Catalyst	B.Emax Cu(II)_s_ ^a^ (eV)	B.Emax Cu(II)_w_ ^b^ (eV)	B.Emax Mn(III) (eV)	B.Emax Mn(IV) (eV)	B.Emax O_L_ (eV)	B.Emax O_ads_ (eV)	$\frac{Mn\left(IV\right)}{Mn\left(III\right)}$	$\frac{Cu}{M}$ ^c^ (Nominal = 0.15)	$\frac{O_{L}}{M}\;$ ^c^ (Nominal = 1.5)
**BMC**	933.1	934.5	641.3	642.3	528.9	530.6	1.3	0.09	0.8
**BMC-Ca**	933.3	934.9	641.4	642.4	528. 9	530.7	1.2	0.07	0.8
**BMC-La**	933.2	935.1	641.3	642.3	528.9	530.7	1.1	0.10	0.9
**BMC-Mg**	933.1	935.0	641.1	642.2	528.7	530.9	1.2	0.09	0.9
**BMC-Sr**	933.2	935.2	641.2	642.3	528.9	530.6	1.3	0.09	0.9
**BMC-Ce**	933.1	934.8	641.3	641.3	528.8	530.5	1.2	0.07	0.9

^a^ s = strong, ^b^ w = weak, ^c^ M = Ba + Mn + Cu.

**Table 6:** T_10%_, T_50%_, and selectivity to CO_2_ (S_CO2_) for soot oxidation of BM and BM-A catalysts.

Catalysts	1% O_2_/He			100% He
	S_CO2_ (%)	T_50%_ (°C)	T_10%_ (°C)	T_10%_ (°C)
**Soot**	44	714	631	-
**BM**	73	710	610	813
**BM-La**	93	708	606	791
**BM-Mg**	92	684	589	813
**BM-Sr**	93	711	591	789
**BM-Ce**	90	641	548	772
**BM-Ca**	91	680	584	823

**Table 7:** T_10%_, T_50%_ and selectivity to CO_2_ (S_CO2_) for soot oxidation in the two tested atmospheres of BMC and BMC-A catalysts.

Catalysts	1% O_2_/He			100% He	
	SCO_2_ (%)	T_50%_ (°C)	T_10%_ (°C)	T_10%_ (°C)	SCO_2_ (%)
**Soot**	44	756	631	-	-
**BMC**	70	732	599	879	33
**BMC-Ca**	93	671	606	859	37
**BMC-La**	94	671	588	611	40
**BMC-Mg**	92	695	605	660	25
**BMC-Sr**	88	712	582	689	27
**BMC-Ce**	94	693	610	646	32

**Figure A1:**
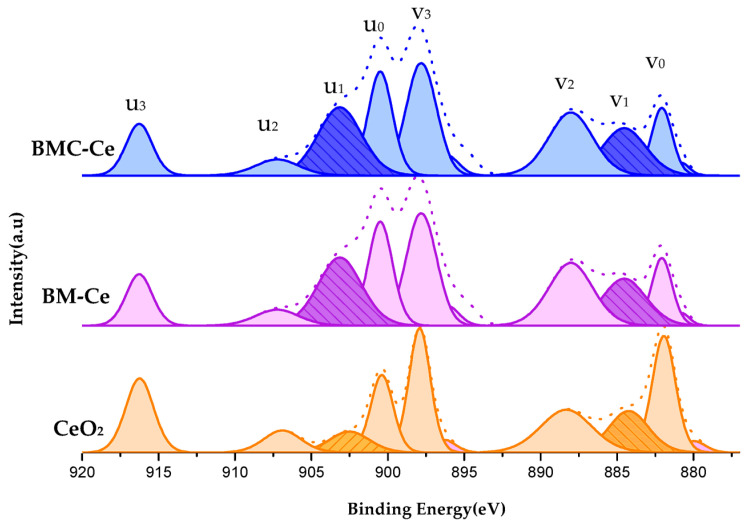
Deconvoluted X-ray photoelectron spectra of Ce 3d core levels for BMC-Ce (blue colour), BM-Ce (purple colour) and CeO_2_ (orange colour).

## Abbreviations

GDI	Gasoline direct injection
PM	Particulate matter
PM2.5	Particulate matter with a diameter of 2.5 μm
GPF	Gasoline particulate filter
TWC	Three-way catalyst
CO_2_RR	CO_2_ reduction reaction
ORR	Oxygen reduction reaction
OER	Oxygen evolution reaction
ICP-OES	Inductively coupled plasma optical emission spectroscopy
XRD	X-ray diffraction
XPS	X-ray photoelectron spectroscopy
H_2_-TPR	Temperature-programmed reduction with H_2_
O_2_-TPD	Temperature-programmed desorption of oxygen
TCD	Thermal conductivity detector
BE	Binding energy
KE	Kinetic energy
TG-MS	Thermal gravimetric mass spectrometry
Soot-TPR	Soot oxidation in temperature-programmed reaction conditions
BET	Brunauer-emmett-teller
ICDD	International centre of diffraction data
O_L_	Lattice oxygen
O_ads_	Adsorbed oxygen species
OSC	Oxygen storage capacity
SCO_2_	Selectivity to CO_2_

## Appendix A

The deconvoluted Ce 3d spectra and the corresponding XPS data for BM-Ce and BMC-Ce catalysts are shown in Figure A1 and Table A1, respectively. The deconvoluted spectra presents eight sub-peaks: the v quadruplets in the Ce 3d_5/2_ area and the u quadruplets in the Ce 3d_3/2_ region. The distinctive identifiers for the Ce(III) oxidation state are two subpeaks, v_1_ and u_1_ (indicated by regions with deeper colours in Figure A1), whereas the other subpeaks are attributed to the Ce(IV) oxidation state [64,65]. The surface Ce(IV) amount in the BM-Ce sample is comparable to that of the CeO_2_ sample used as a reference, while the BMC-Ce sample exhibits a larger abundance of Ce(III). This implies that the presence of Ce(III) species seems to be favoured for the copper-containing sample.

Ce(III) = u_1_ + v_1_ (A1)

Ce(IV) = u_0_ + u_2_ + u_3_ + v_0_ + v_2_ + v_3_ (A2)

**Table A1:** XPS data of CeO_2_, BM-Ce, and BMC-Ce catalysts.

Catalyst	CeO_2_	BM-Ce	BMC-Ce
** $\frac{Ce\left(III\right)}{Ce\left(IV\right)}$ **	0.54	0.93	1.33
